# Serum DBI and biomarkers of neuroinflammation in Alzheimer’s disease and delirium

**DOI:** 10.1007/s10072-020-04608-x

**Published:** 2020-07-23

**Authors:** Elisa Conti, Simona Andreoni, Davide Tomaselli, Benedetta Storti, Francesco Brovelli, Roberto Acampora, Fulvio Da Re, Ildebrando Appollonio, Carlo Ferrarese, Lucio Tremolizzo

**Affiliations:** 1 School of Medicine and Surgery and Milan Center for Neuroscience (NeuroMI), University of Milano-Bicocca, Room 2043, Building U8, via Cadore 48, 20900 Monza, MB Italy; 2 Neurology Unit, “San Gerardo” Hospital, Monza, Italy

**Keywords:** Diazepam binding inhibitor, Alzheimer’s disease, Delirium, Serum, Cytokines, Monocytes

## Abstract

**Background:**

Alzheimer’s disease (AD) patients often express significant behavioral symptoms: for this reason, accessible related biomarkers could be very useful. Neuroinflammation is a key pathogenic process in both AD and delirium (DEL), a clinical condition with behavioral symptoms resembling those of AD.

**Methods:**

A total of *n* = 30 AD patients were recruited together with *n* = 30 DEL patients and *n* = 15 healthy controls (CTRL). Serum diazepam binding inhibitor (DBI), IL-17, IL-6, and TNF-α were assessed by ELISA.

**Results:**

DBI serum levels were increased in AD patients with respect to CTRL (+ 81%), while DEL values were 70% higher than AD. IL-17 was increased in DEL with respect to CTRL (+ 146%), while AD showed dispersed values and failed to reach significant differences. On the other hand, IL-6 showed a more robust increase in DEL with respect to the other two groups (+ 185% and + 205% vs. CTRL and AD, respectively), and TNF-α failed to show any change.

**Conclusions:**

DBI may be a very promising candidate for AD, perhaps marking psychomotor DEL-like symptoms, in view of developing future helping tool for practicing physicians. Furthermore, DBI rise in DEL offers novel cues for a better comprehension of the pathogenesis of this potentially fatal condition.

## Introduction

Alzheimer’s disease (AD) is a relentless neurodegenerative dementia characterized by the significant expression of behavioral symptoms (BPSD), often representing the biggest problem in patient management [1]. In particular, some BPSD, such as the psychomotor cluster of agitation/aggression, irritability, and aberrant motor behavior [2], might be extremely disruptive for caregiver home-based management and often require drug treatment. One major problem for practicing physicians when facing this complex situation is represented by a potential reporting bias, since BPSD expression and severity are mainly detected through caregivers’ impressions. For this reason, the identification of accessible biomarkers could be extremely useful, and serum/blood-based attempts have already been made [3]. Feasible biomarkers should also be chosen among those reflecting core processes of the underlying pathologies [4].

Among different pathological processes involved in AD, neuroinflammation is especially interesting when looking for a marker of the above quoted psychomotor cluster, due to the clinical resemblances to two conditions, also characterized by the rise of peripheral inflammatory markers: sickness behavior [5] and —in particular—delirium (DEL). This latter condition, in fact, is strictly intertwined to dementia, since AD patients very often develop DEL when facing a psychophysical stress (e.g., hospitalization, infection), and cognitively spared DEL patients are at great risk of developing dementia in the following few months [6], clearly involving both reduced neuronal reserve and *inflammaging* as key pathological processes [7]. In fact, when considering DEL pathophysiology, inflammatory and neuroendocrine abnormalities are clearly present, with benzodiazepines (BDZ) acting often as supplementary precipitants in predisposed patients. Several reports already indicate that numerous inflammatory cytokines and different chemokines are robustly increased in serum/plasma [8] and CSF [9] of DEL patients, often correlating with DEL duration, severity, and mortality [10].

Ideal peripheral markers of neuroinflammation should, in any case, display a central role as well, in order to directly account for possible phenotypic variability. Diazepam binding inhibitor (DBI; [11]) is a very interesting candidate since, as an endozepine, it binds to the peripheral BDZ receptor (known as TSPO, the imaging marker of activated microglia and neuroinflammation [12]), initiating the biosynthesis of neurosteroids and promoting monocyte chemotaxis [13]. Furthermore, besides this peripheral role, DBI also works centrally as the allosteric modulator of the GABA-A receptor on the BDZ binding site [14]: for this reason, it has been involved, with contrasting evidence, in disorders characterized by anxiety expression [14, 15].

Considering this role of DBI, in this exploratory work, we decided to assess DBI serum levels in AD patients with respect to DEL ones and comparable healthy controls, searching for a putative “core biomarker” of the common physiopathological link of neuroinflammation. In order to speculate on the possible specificity of the DBI findings, we also included on the same samples the assessment of three neuroinflammation biomarkers, chosen considering the biological roles of DBI and keeping in mind our ultimate focus on BPSD. More specifically, we included interleukin-17 (IL-17), which is a chemokine as DBI, and the two classical inflammatory cytokines tumor necrosis factor-α (TNF- α) and IL-6 (this latter a myokine as well, possibly marking behavioral symptoms belonging to the psychomotor cluster [3]).

## Results

**Fig. 1:**
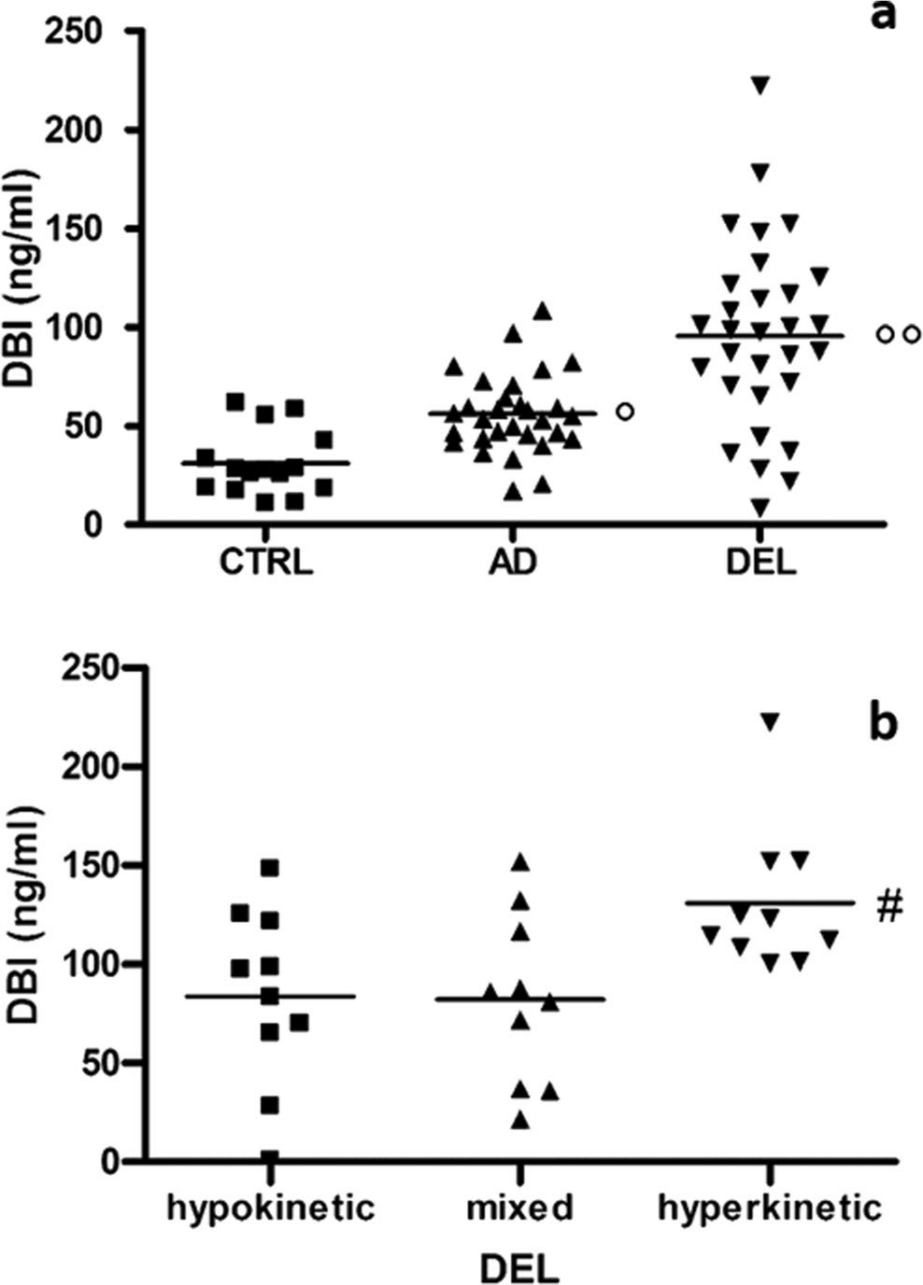
Serum DBI. **a** *p* < 0.0001 at ANOVA, followed by Newman-Keuls multiple comparison test, *p* < 0.05 vs. CTRL, *p* < 0.001 vs. both CTRL and AD. **b** *p* < 0.03 at ANOVA, followed by Newman-Keuls multiple comparison test, #*p* < 0.05 vs. both hypokinetic and mixed. Mean values are shown

DBI serum levels were increased in AD patients with respect to CTRL (56.3 ± 20.1 vs. 31.1 ± 16.5 ng/ml, respectively, + 81%, *p* < 0.05), while DEL values were 70% higher than AD (95.8 ± 47.7 ng/ml, *p* < 0.001) and more than threefold higher with respect to CTRL (*p* < 0.001) (Fig. 1a). When considering DEL subtypes, DBI showed ~ 60% higher values in hyperkinetic patients with respect to the other two groups (130.9 ± 36.9 vs. 82.3 ± 42.9 vs. 83.9 ± 45.0 ng/ml, hyperkinetic vs. mixed vs. hypokinetic, respectively, *p* < 0.05; Fig. 1b). No other differences were shown when considering all the available clinical and demographic factors.

**Fig. 2:**
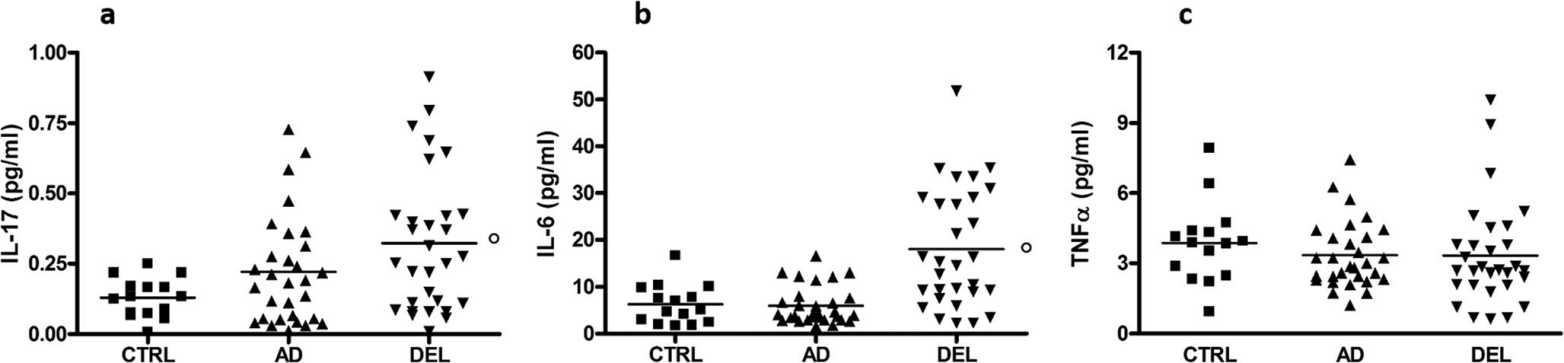
Serum levels of **a** IL-17, *p* < 0.01 at ANOVA, followed by Newman-Keuls multiple comparison test, *p* < 0.01 vs. CTRL. **b** IL-6, *p* < 0.0001 at ANOVA, followed by *p* < 0.001 vs. both AD and CTRL. **c** TNF-α, *p* = 0.60 at ANOVA. Mean values are shown

IL-17 increased more than twofold in DEL with respect to CTRL (246%, 0.32 ± 0.25 vs. 0.13 ± 0.07 pg/ml, respectively, *p* < 0.01), while AD showed dispersed values (0.22 ± 0.19 pg/ml) and failed to reach significant differences with respect to the other two groups (Fig. 2a). On the other hand, IL-6 showed a more robust increase (about threefold) in DEL with respect to the other two groups (18.0 ± 12.7 vs. 5.9 ± 4.0 vs. 6.3 ± 4.2 pg/ml, DEL vs. AD vs. CTRL, respectively, *p* < 0.001; Fig. 2b), and TNF-α failed to show any change (3.3 ± 2.2 vs. 3.3 ± 1.4 vs. 3.8 ± 1.7 pg/ml, DEL vs. AD vs. CTRL, respectively; Fig. 2c). IL-17, IL-6, and TNF-α failed to show significant differences among the three DEL subtypes (data not shown). Finally, no correlations emerged among all the quantified serum biomarkers.

## Materials and methods

### Subject recruitment

Following ethical approval by our hospital committee, *n* = 30 AD patients (M/F 14/16, age 80.1 ± 5.3 years old, MMSE 17.5 ± 5.7, disease duration 51.1 ± 29.1) and *n* = 30 age- and sex-matched delirium patients (DEL, M/F 14/16, age 79.2 ± 7.8 years old) were recruited, together with *n* = 15 age- and sex-comparable healthy controls (CTRL, M/F 7/8, age 79.0 ± 6.1 years old, MMSE always > 26, and negative history for neurological and/or psychiatric disorders and/or symptoms). AD and DEL patients were recruited if (a) a legal representative was available or (b) if they were able to express informed consent (this condition was confirmed by th*e* MacArthur Competence Assessment Tool [16]), or (c) only for DEL patients, if their clinical condition clearly reverted. Probable AD outpatients were recruited according to the NINCDS-ADRDA criteria and alternative diagnoses were excluded by MR brain imaging and an extensive neuropsychological test battery. Albeit amyloid biomarkers were unavailable for this study, MR atrophy of the hippocampal regions and/or bilateral parietal ^18^F-FDG PET hypometabolism was documented for all AD patients. DEL patients were initially selected according to DSM-V criteria. Furthermore, DEL was defined also by clinical judgment (considering the frequent presence of pre-existing confounders such as dementia [17] and if the 4AT was ≥ 6; Table 1). DEL patients were recruited in order to equally represent each subtype, i.e., *n* = 10 mixed subtype, *n* = 10 hyperkinetic subtype, *n* = 10 hypokinetic subtype. DEL predisposing factors and the presence of pre-existing dementia are shown in Table 1, together with serum sodium, potassium, and ammonia assessed by our hospital laboratory for clinical purposes (mostly within normal levels).

**Table 1:** DEL predisposing factors and the presence of pre-existing dementia. *SOL*, space-occupying lesions (tumors); *CVD*, cerebrovascular diseases (ischemic)

	Delirium, *n* = 30
Predisposing factors	Infectious, *n* = 5 (16.7%)
	SOL, *n* = 4 (13.3%)
	CVD, *n* = 19 (63.3%)
	Other, *n* = 2 (6.7%)
Pre-existing dementia, *n* (%)	17 (56.7%)
Sodium, mM	141.2 ± 2.9 (136–147)
Potassium, mM	4.1 ± 0.4 (3.2–4.9)
Ammonia, μg/100 ml	43.2 ± 23.3 (18–69)
4AT	8.8 ± 2.2 (6–12)

### Quantification of serum markers

Coded blood samples were obtained after overnight fasting and immediately centrifuged: serum aliquots were frozen at − 80 °C until blind assessment. All targets were measured in triplicate by commercial ELISA kit: DBI (sensitivity: mean minimum detectable dose (MDD) 25 pg/ml, AbFRONTIERS, Seoul, Korea), IL-17 (sensitivity: MDD 0.017–0.051 pg/ml, mean MDD 0.029 pg/ml), IL-6 (sensitivity: MDD 0.016–0.110 pg/ml, mean MDD 0.039 pg/ml), and TNF-α (sensitivity: MDD 0.011–0.049 pg/ml, mean MDD 0.022 pg/ml) (Quantikine® Colorimetric Sandwich ELISA Kits, R&D Systems, Minneapolis, U.S.).

### Statistical analysis

Data are shown as mean ± SD. ANOVA followed by Newman-Keuls Multiple Comparison post hoc test was used for assessing differences among the study groups. Two-tailed Pearson’s *r* test was used for correlative analyses.

## Discussion

We assessed a panel of neuroinflammatory markers comparing AD and DEL patients to healthy controls. Serum DBI was specifically chosen since this endozepine displays unique features: it has been characterized as the endogenous ligand of the TSPO, a well-known marker of the activated monocyte/microglia [14]. In fact, TSPO activation promotes chemotaxis in peripheral monocytes [13], a pivotal process sustaining neuroinflammation: protein aggregates and chemokines both recruit these cells within the central nervous system [18]. Following this step, peripheral monocytes change conformation, giving rise to the blood-born macrophage, a subpopulation of microglia actively participating to the neuroinflammatory process [19]. A change in DBI concentrations could then promote the process of chemotaxis and subsequent neuroinvasion, as already proposed for the classical chemokine MCP-1 [20]. Moreover, TSPO activation starts the process of neurosteroid biosynthesis at the level of mitochondria [21]. Neurosteroids, or in this case, perhaps more properly, neuroactive steroids, display pleiotropic properties, promoting neurotrophin synthesis [22] and acting on receptors expressed both on the neuronal membrane and directly on the DNA [23]. For these reasons, TSPO and neurosteroids have already been considered with interest in the field of psychiatry [21, 24]. Finally, DBI acquires further importance as potential biomarker of those conditions expressing behavioral symptoms, since it is able to interact with the central BDZ binding site, on the GABAergic receptors. This direct central role offers a supplementary potentially deranged mechanism in those conditions expressing behavioral symptoms, such as AD or schizophrenia [25, 26]. Our data show that serum DBI was robustly increased in AD patients with respect to controls and displayed a further increase in DEL patients. In fact, 80% of DEL patients displayed serum DBI levels above 2SD of controls, and ~ 50% above 2SD of AD patients.

On the other hand, none of the three chosen inflammatory cytokines displayed a similar pattern, since IL-17 and IL-6 were significantly increased only in DEL patients and TNF-α failed to show any change among the three groups. IL-17 is a pro-inflammatory lymphatic chemokine produced by a subset of T helper cells, activating a cascade that leads to the secretion of chemokines, recruiting both monocytes and neutrophils to the inflammation site. Albeit previous studies failed to report an increase in those patients developing DEL after cardiac surgery [27] or in concomitance to septic shock [28], here we report a ~ 2.5-fold increase versus controls. In any case, AD patients displayed largely variable results that led to the lack of significant changes with respect to the other two groups. Possibly, this result should be reviewed increasing sample size in order to minimize potential analytical confounders and in order to test the possibility of stratifying the behavioral phenotypes expressed by AD patients. IL-6 is a classical inflammatory cytokine produced by immune cells and a myokine; i.e., it is also produced by muscle cells during contraction. This dual role is extremely interesting when thinking to explore behavioral phenotypes associated to psychomotor activation, as already proposed [3], albeit we failed to report any change in AD patients. On the other hand, as previously reported [29], IL-6 serum levels in DEL patients were significantly increased, being ~ 50% of the time above 2SD of both AD and control values. However, IL-6 displayed a significant variability of the detected values (coefficient of variation, CV = ~ 0.70), with respect to DBI (CV between 0.35 and 0.50). Given these results, we are now planning to measure DBI serum levels in a large number of AD patients in order to post hoc stratify them according to the expressed behavioral dysfunctions, with special attention to those strictly resembling DEL, and especially the hyperkinetic form of this condition. One limit of the present study that we are currently addressing is the lack of determination of amyloid presence. Furthermore, one interesting arising question regards the source of this elevated serum peptide. In fact, DBI is produced by peripheral organs, such as the liver or steroidogenic tissues, but also by cells within the central nervous system [30]. Our preliminary data shows that DBI levels in AD patients are of about the same order of magnitude, but 50% higher in CSF with respect to cognate serum samples, with a linear correlation between the two compartments (unpublished observations). This further supports the idea that peripheral monocytes could be realistically recruited toward the central compartment, fueling neuroinflammation.

In conclusion, DBI may be a very promising candidate for marking the psychomotor cluster of BPSD in AD patients, perhaps offering in the future a valuable helping tool to practicing physicians. As a final point, the DBI rise in DEL offers novel cues for a better comprehension of the pathogenesis of this potentially fatal condition.

## Data Availability

Data and material are available at the Laboratory of Neurobiology, School of Medicine and Surgery, University of Milano-Bicocca, Monza, Italy.
